# Comparing the Clinical and Radiographic Outcomes of Humeral Shaft Fractures by Treatment Type

**DOI:** 10.7759/cureus.58658

**Published:** 2024-04-20

**Authors:** Chrystina L James, Jager Haan, Susan G Wager, Yash Hegde, Trevor D Wolterink, Stephanie Muh

**Affiliations:** 1 Department of Orthopaedic Surgery, Henry Ford Health System, Detroit, USA; 2 Department of Orthopaedic Surgery, Michigan State University College of Human Medicine, East Lansing, USA; 3 Department of Orthopaedic Surgery, Wayne State University School of Medicine, Detroit, USA

**Keywords:** nonoperative, operative, humeral shaft, fracture, humerus

## Abstract

Purpose: Humeral shaft fractures are common orthopedic injuries, representing 1-5% of all fractures. There is conflicting literature regarding the superiority of operative versus nonoperative treatment of these fractures. The purpose of this study was to examine functional outcomes and time to radiographic union in humeral shaft fractures with the hypothesis that both would be improved in patients treated operatively relative to those treated nonoperatively.

Methods: This retrospective cohort study examined patients with humeral shaft fractures treated at a single large healthcare system between 2010 and 2020. A chart and radiograph review were performed to collect information on demographics, fracture, treatment, and outcome information. These measures were compared between patients treated operatively and nonoperatively.

Results: Five hundred seventeen adult patients meeting inclusion criteria were identified; 233 were treated nonoperatively, and 284 were treated operatively. The mean patient age was 50.2 years in those who underwent surgery relative to 59.9 years in those treated without surgery (P<0.001). Operatively-treated patients had significantly faster time to radiographic union at a median of 113 days compared to a median of 161 days in nonoperatively-treated patients (P=0.001). The operative group was made weight-bearing as tolerated significantly faster than the nonoperative group (84 days versus 98 days, respectively, P=0.002). No statistically significant difference was seen between the two treatment groups in rates of complications or range of motion at the time of radiographic union. However, patients who underwent surgery were found to be up to two times more likely to achieve full shoulder forward elevation by the time of their final follow-up than those treated without surgery (P=0.011).

Conclusion: Patients with humeral shaft fractures treated operatively have faster time to union, earlier weight bearing, and no change in the rate of complications compared to patients treated nonoperatively.

## Introduction

Humeral shaft fractures are common orthopedic injuries, representing 1% to 5% of all fractures [1]. Historically, nonoperative management consisting of functional bracing, splinting, or casting has been the gold standard treatment. However, the last several decades have seen a steady rise in the rate of operative intervention [2-5]. This trend toward surgical intervention is supported by several recent prospective cohort studies showing higher union rates and improved outcomes in patients treated operatively [6,7].

Outside of these few recent studies, most previous research examining the treatment of humeral shaft fractures does not clearly indicate the superiority of one treatment over another. Several studies comparing operative and nonoperative management show no difference in ultimate range of motion (ROM) or time to a clinical union, which is commonly defined as the absence of pain and motion at the fracture site. However, higher rates of nonunion and malunion have been seen in patients treated nonoperatively [6,8-10]. Additionally, there is limited data showing overall similar functional outcomes in patients treated operatively and nonoperatively [6,11]. While the majority of patients are able to return to work or sport following humeral shaft fractures, it is unclear if surgical management affects the likelihood or timing of this [12]. Despite numerous studies examining rates of union and time to clinical union, the authors are unaware of any studies comparing time to radiographic union between operative and nonoperative treatment.

The primary aim of this study was to compare the time to achieving radiographic union and being made weight-bearing as tolerated (WBAT) following operative and nonoperative management of humeral shaft fractures. We hypothesized that the times to radiographic union and WBAT would be faster in patients treated operatively than in those treated nonoperatively. Secondary outcomes examined included ROM following operative and nonoperative treatment, reoperation rates, conversion to operative treatment, and complication rates between treatment groups.

This article was previously presented as a meeting abstract at the American Academy of Orthopaedic Surgeons annual meeting in March 2023 as well as at the Mid-America Orthopaedic Association annual meeting in April 2023.

## Materials and methods

This retrospective cohort study was performed at a single healthcare system with five hospitals in both urban and suburban settings, ranging from a Level I trauma center to a non-trauma hospital. All humeral shaft fractures treated between 2010 and 2020 were identified using the International Classification of Diseases, Ninth Revision, Clinical Modification (ICD-9-CM) codes, ICD-10-CM codes, and Current Procedural Technology (CPT) codes. Patients were included if they had an ICD-9 code of 812 (fracture of humerus), an ICD-10 code of S42.3 (fracture of shaft of humerus), or CPT codes 24500, 24505, 24515, or 24516 (closed treatment of humeral shaft fracture without manipulation, closed treatment of humeral shaft fracture with manipulation, open treatment of humeral shaft fracture with plates/screws, or treatment of humeral shaft fracture with insertion of an intramedullary implant, respectively). This identified 1917 unique patients. This study was reviewed and approved by the Institutional Review Board of Henry Ford Health System (approval number: 14031). This research did not receive any specific grant from funding agencies in the public, commercial, or not-for-profit sectors.

Patients were excluded if they were less than 18 years old at the time of injury. Patients with bilateral, pathologic, periprosthetic, humeral neck, and intra-articular fractures were also excluded, as were those with a history of a previous ipsilateral humeral shaft fracture. This yielded a sample size of 517 patients. Of the 517 patients identified, 79 patients had no follow-up information recorded. These patients were included in the analysis of patient demographics, fracture characteristics, and initial treatment but were excluded from the outcomes analysis.

A review of patient charts and radiographs was utilized to obtain information on demographics, fracture characteristics, treatment, follow-up, and outcomes. Nonoperative treatment consisted of immobilization in a Sarmiento brace for a duration determined by the treating surgeon. Operative treatment consisted of either open reduction internal fixation (ORIF) with a plate and screws or intramedullary nailing (IMN). The date of fracture care was recorded as the date of surgery for patients treated operatively and the date of the first orthopedic clinic appointment for those treated without surgery. Radiographic union was defined as a bridging callus seen on three cortices and was determined by assessing orthogonal radiographs of the humerus taken at routine follow-up appointments. All radiographs were analyzed by the same member of the study team.

Length of follow-up, time to radiographic union, and time to WBAT were calculated as the number of days from the date of fracture care to the date of the respective event. In the event that patients were initially treated nonoperatively and subsequently converted to operative treatment, the date of initial nonoperative fracture care was utilized for these calculations. Other measures collected were the Visual Analogue Scale (VAS) pain score on a scale of 0-10, occurrence of any treatment-related complication, conversion to operative treatment, need for reoperation, and ROM at radiographic union and final follow-up.

Categorical variables are presented as frequencies and percentages; means and standard deviations are presented for normally distributed continuous variables. For non-normally distributed continuous variables, medians and the first and third quartiles are presented. Comparisons between the treatment groups for categorical variables are performed using chi-square tests, while Fisher’s exact test is used when expected cell counts are <5. For continuous variables (excluding time to union and WBAT variables), two-group comparisons are performed using independent two-sample t-tests if the variable is normally distributed and using Wilcoxon rank sum tests if the variable is non-normally distributed. For the time to radiographic union and time to WBAT, Kaplan-Meier estimation using the log-rank test is used to compare the treatment groups. Poisson regression with robust error variance was used to determine whether the risk of achieving each ROM at the final follow-up differs by treatment when controlling for the length of follow-up. Statistical significance is pre-specified at p<0.05. All analyses are performed using SAS 9.4 (SAS Institute Inc., Cary, NC, USA).

## Results

Demographics of cohorts

In the 10-year study period, 517 adult patients with unilateral humeral shaft fractures were identified with demographics as shown in Table 1. The patients were 59.2% female and predominantly Caucasian (61.7%) and African American (31.3%), with an average age of 54.6 years and a median BMI of 28.3. The majority of patients were nonsmokers (72.3%) and employed (54.7%). The most common insurance coverage was Medicare or Medicaid (62.7%).

**Table 1:** Demographics of study population A: Median and the first and third quartiles are presented due to skewed BMI data within this sample

Variable	Response	Overall	Nonoperative	Operative	p-value
Age (years)	Mean ± SD	54.6 ± 21.4	59.9 ± 20.9	50.2 ± 20.9	<0.001
Gender (n)	Female	306 (59.2%)	153 (65.7%)	153 (53.9%)	0.007
	Male	211 (40.8%)	80 (34.3%)	131 (46.1%)	
Race (n)	Caucasian	319 (61.7%)	144 (64.3%)	175 (62.9%)	0.195
	African American	162 (31.3%)	67 (29.9%)	95 (34.2%)	
	Other	21 (4.1%)	13 (5.8%)	8 (2.9%)	
	Not reported	14 (2.7%)	-	-	
Marital status (n)	Single	216 (41.8%)	82 (36.3%)	134 (49.4%)	0.024
	Married	169 (32.7%)	90 (39.8%)	79 (29.2%)	
	Divorced	74 (14.3%)	19 (8.4%)	19 (7.0%)	
	Widowed	38 (7.4%)	35 (15.5%)	39 (14.4%)	
	Not reported	20 (3.9%)	-	-	
BMI^A^	Median (Q1, Q3)	28.3 (23.8, 34.3)	27.5 (23.3, 33.2)	29 (24.6, 35.0)	0.048
Hand dominance (n)	Right	419 (81.0%)	185 (91.6%)	234 (93.6%)	0.413
	Left	33 (6.4%)	17 (8.4%)	16 (6.45)	
	Not reported	65 (12.6%)	-	-	
Smoking status (n)	No	374 (72.3%)	174 (75.0%)	200 (71.4%)	0.365
	Yes	138 (26.7%)	58 (25.0%)	80 (28.6%)	
	Not reported	5 (1.0%)	-	-	
Insurance (n)	Medicare or Medicaid	324 (62.7%)	163 (70.3%)	183 (65.4%)	0.47
	Auto or worker compensation	22 (4.2%)	Combined with the above value	Combined with the above value	
	Private	150 (29.0%)	63 (27.2%)	87 (31.1%)	
	Uninsured	16 (3.1%)	6 (2.6%)	10 (3.6%)	
	Not reported	5 (1.0%)	-	-	
Employment status (n)	Unemployed	234 (45.3%)	123 (65.8%)	111 (45.5%)	<0.001
	Employed	197 (38.1%)	64 (34.2%)	133 (54.5%)	

Of the 517 patients identified, 54.9% received operative treatment at an average of three days from the date of injury, and 45.1% received nonoperative treatment. Of those treated operatively, 90.8% were treated with ORIF, and 9.2% were treated with IMN. Patients treated operatively were significantly younger than those treated nonoperatively, with an average age of 50.2 years compared to 59.9 years, respectively (P<0.001). Nearly two-thirds of those treated nonoperatively were female (65.7%), compared to about half of the operative group (53.9%) (P=0.007). The rate of employment was higher amongst operative patients than nonoperative patients at 54.5% and 34.2%, respectively (P<0.001). Operative patients had a statistically significant but clinically irrelevant difference in body mass index, with a median of 29 compared to 27.5 in nonoperative patients (P=0.048). There were also significant differences in the marital status of the groups, with single patients making up the largest portion of patients treated operatively (P=0.024) (Table 1).

Fracture characteristics

The most common mechanisms of injury overall were ground-level falls (59.6%), motor vehicle accidents (MVA) (15.9%), and gunshot wounds (GSW) (6.6%). There were 47 open fractures (9.1%), with Gustilo Anderson type 1 being the most common (55.3% of all open fractures). Fractures were predominantly in the patient’s non-dominant arm (59.2%). Around half of all fractures were located in the middle third of the humeral diaphysis (53.6%), and 48 patients (9.3%) had an associated radial nerve palsy on initial examination. In 423 patients (81.8%), the humeral shaft fracture was an isolated injury (Table 2).

**Table 2:** Fracture characteristics - overall MVA: motor vehicle accidents, GSW: gunshot wounds, ORIF: open reduction internal fixation, IMN: intramedullary nailing

Variable	Response	N (%)
Mechanism	Ground level fall	308 (59.6%)
	MVA	82 (15.9%)
	Other	37 (7.2%)
	GSW	34 (6.6%)
	Fall from height	23 (4.4%)
	Assault	20 (3.9%)
	Fall down stairs	13 (2.5%)
Location (diaphyseal third)	Proximal	125 (24.2%)
	Mid	277 (53.6%)
	Distal	115 (22.2%)
Polytrauma	No	423 (81.8%)
	Yes	94 (18.2%)
Dominant hand?	No	306 (59.2%)
	Yes	211 (40.8%)
Open or closed?	Closed	470 (90.9%)
	Open	47 (9.1%)
Gustilo-Anderson classification	1	26 (55.3%)
	2	14 (29.8%)
	3	7 (14.9%)
Nerve palsy	None	462 (89.7%)
	Radial	48 (9.3%)
	Other	5 (1.0%)
Treatment	Operative	284 (54.9%)
	Nonoperative	233 (45.1%)
Type of operative treatment	ORIF	258 (90.8%)
	IMN	26 (9.2%)

As shown in Table 3, of fractures treated nonoperatively, 66.1% were middle-third diaphysis, 24.5% were proximal-third diaphysis, and 9.4% were distal-third diaphysis. This was significantly different than those treated operatively, which were 43.3% middle-, 23.9% proximal-, and 32.7% distal-third diaphysis (P<0.001). The mechanism of injury differed between cohorts; there were significantly more ground-level falls in patients treated nonoperatively compared to those treated operatively, 75% of patients relative to 46.8%, respectively (P<0.001). The humeral shaft fracture was an isolated injury in 72.5% of patients treated operatively, compared to 93.1% of patients treated nonoperatively (P<0.001).

**Table 3:** Fracture characteristics by treatment cohort

Variable	Response	Nonoperative, n (%)	Operative, n (%)	p-value
Mechanism	Ground level fall	175 (75.1%)	133 (46.8%)	<0.001
	MVA	18 (7.7%)	64 (22.5%)	
	Other	40 (17.2)	87 (30.8%)	
Location (diaphyseal third)	Proximal	57 (24.5%)	68 (23.9%)	<0.001
	Mid	154 (66.1%)	123 (43.3)	
	Distal	22 (9.4%)	93 (32.7%)	
Polytrauma	No	217 (93.1%)	206 (72.5%)	<0.001
	Yes	16 (6.9%)	78 (27.5%)	

Outcomes

As stated previously, of the 517 patients identified for this study, 79 had no follow-up after their initial injury and/or surgery and were excluded, resulting in 438 patients included in the outcomes analysis. Information on the date of radiographic union was available for 273 patients (62.3%), and the date of WBAT was available for 338 patients (77.3%). The time from the date of fracture care to the date of radiographic union was a median of 161 days in patients treated nonoperatively, significantly higher than in patients treated operatively with a median of 113 days (P=0.001). The time from the date of fracture care to the date of WBAT was also significantly greater in the nonoperative treatment group at a median of 98 days relative to the operative treatment group at a median of 84 days (P=0.002). Hazard ratio analysis showed that at any particular point in time, the operative group was 1.53 times more likely to have reached radiographic union than the nonoperative group (95% CI (1.20, 1.95), P=0.001). At any particular point in time, the operative group was also 1.40 times more likely to have reached WBAT than the nonoperative group (95% CI (1.13, 1.74), P=0.002).

Complication rates were not significantly different between the operative and nonoperative cohorts, nor were pain scores at the last follow-up. The length of follow-up was similar in both cohorts. Nineteen point three percent of patients initially treated nonoperatively were converted to operative treatment at a mean of 110 days from the date of injury. Eleven point one percent of those initially treated operatively went on to require another surgery, and 30% of these reoperations were for the removal of hardware (Table 4).

**Table 4:** Outcomes by treatment group A: Median and the first and third quartiles are presented due to skewed data

Variable	Response	Nonoperative, n (%)	Operative, n (%)	p-value
Time from date of fracture care to date of last follow-up^A^	Median (Q1, Q3)	131 (77,227)	131 (72,272)	0.771
Complications	No	139 (72.8%)	197 (79.8%)	0.086
	Yes	52 (27.2%)	50 (20.2%)	
Operative only: need for reoperation?	No	N/A	216 (88.9%)	N/A
	Yes	N/A	27 (11.1%)	
Nonoperative only: conversion to operative treatment	No	151 (80.7%)	N/A	N/A
	Yes	36 (19.3%)	N/A	
Any pain last follow-up? (0 vs. 1)	No (pain=0)	98 (57.3%)	116 (53.7%)	0.479
	Yes (pain=1)	73 (42.7%)	100 (46.3%)	

ROM data was also analyzed when available; however, this information was only available to a minority of patients. There were no statistically significant differences between treatment groups at the time of radiographic union in the percentage of patients achieving full shoulder abduction or forward elevation (classified as more than 160 degrees for the purposes of this study). There were also no differences in the number of patients achieving full elbow extension (0-5 degrees), full elbow flexion (greater than 120 degrees), or a functional elbow ROM (greater than 100-degree arc of motion).

In looking at these same ROM measurements throughout the follow-up period and adjusting for length of follow-up, patients who underwent operative treatment were up to two times more likely to achieve full shoulder forward elevation by final follow-up compared to the nonoperative group (RR = 1.35 (95% CI (0.96, 1.91)), P=0.011). There were no other significant differences in ROM at the final follow-up, as shown in Table 5.

**Table 5:** Effect of treatment group on the risk of achieving ROM at final follow-up (while controlling for length of follow-up) ROM: range of motion

Outcome	Comparison	Risk ratio (95% confidence interval)	p-value
Shoulder abduction >160 degrees	Operative vs. nonoperative	1.35 (0.96, 1.91)	0.084
Shoulder forward elevation >160 degrees	Operative vs. nonoperative	1.48 (1.09, 2.00)	0.011
Elbow extension 0-5 degrees	Operative vs. nonoperative	0.97 (0.81, 1.16)	0.744
Elbow flexion >120 degrees	Operative vs. nonoperative	1.07 (0.90, 1.27)	0.418
Elbow functional arc of motion >100 degrees	Operative vs. nonoperative	1.03 (0.88, 1.19)	0.734

## Discussion

This study shows that operative treatment of a humeral shaft fracture results in significantly shorter times to radiographic union and weight bearing compared to nonoperative treatment without a difference in the rate of complications.

Nonoperative management has historically been the gold standard for treating humeral shaft fractures [3]. It is inexpensive as a treatment and avoids the risks and complications inherent to surgical management, such as postoperative infections, iatrogenic nerve palsies, and complications related to anesthesia [6,13]. However, in considering lost wages, productivity, and workplace absences, nonoperative treatment may actually be a higher cost to society if patients treated nonoperatively remain unable to work for a longer period of time [14]. Additionally, some studies demonstrate high rates of non-union and malunion with nonoperative treatment, which may ultimately result in a later return to function, increased complications, and the ultimate need for surgical correction [8-11,15]. While many existing studies have compared the rate of union between different treatment modalities, we are unaware of others examining the time to radiographic union.

While the existing literature examining functional outcomes following humeral shaft fractures is limited, the majority of studies have not shown significant differences in these outcomes between patients treated with or without surgery. To our knowledge, time to WBAT has not frequently been studied as a metric in comparing treatment outcomes for humeral shaft fractures. Time to WBAT is a well-established method to quantify outcomes in lower extremity injuries such as ankle and hip fractures. In these injuries, earlier time to WBAT has been correlated with improved outcomes and a quicker return to activity. However, it has not commonly been used in upper extremity injuries [16-18]. In this study, patients treated with surgery achieved WBAT significantly faster than patients treated without surgery. Earlier weight bearing may be indicative of an earlier return to work and ability to perform ADLs independently, as well as overall improved outcomes related to functional status.

Another measure of functional status is the ROM. There is very little literature examining ROM following humeral shaft fractures, but patients achieving full ROM may be more likely to return to their pre-injury activities and to be able to complete ADLs independently. One recent prospective cohort study examined elbow ROM and found no differences based on treatment; another recent study examined shoulder and elbow ROM and found improved ROM results in patients treated operatively [6-7]. Our study found that achieving full shoulder forward elevation was significantly more likely in patients treated operatively than in those treated nonoperatively. However, it is important to recognize that less than half of the patients in either group were able to achieve full forward elevation or abduction. Thus, although patients treated operatively may be more likely to regain normal ROM than those treated nonoperatively, the overall likelihood of achieving normal motion after a humeral shaft fracture may be low and is important to counsel patients on. The existing data on ROM as an outcome in humeral shaft fractures is still very limited, and further studies are needed to reach any definitive conclusions.

In addition to differences seen in outcomes between treatment groups, there were also several differences noted in patient and fracture characteristics. Patients in the operative group were significantly younger, which may represent some selection bias. It is possible that older patients had a lower baseline functional status or had more medical comorbidities and thus were at a higher surgical risk, making them less likely to be offered surgery. They also may have been more likely to have low-energy mechanisms of injury, resulting in less fracture displacement and fewer concomitant injuries [19]. The operative group was also more likely to be male, employed, and single than the nonoperative group. This may be due to a perception among patients that operative treatment will allow them to return to work and activity faster. Additionally, patients treated operatively had higher rates of high-energy mechanisms and polytraumatic injuries, which may indicate more displaced or comminuted fractures. Previous studies have shown that increased fracture angulation and displacement correlate with higher rates of nonunion in patients treated nonoperatively; some surgeons suggest that fracture angulation is a relative indication for surgical fixation [20].

There are several limitations to this study. The primary limitation is the retrospective study design. As such, variability exists in the length of follow-up, frequency of appointments, surgeon training and experience, operative technique, documentation, and treatment, as well as postoperative and rehabilitation protocols. Although the length of follow-up between operative and nonoperative treatments was not significantly different, there was individual variability between patients, which may have resulted in complications and outcomes that were not documented. There may also be selection bias regarding which patients were offered surgical intervention, resulting in younger, more active, and/or healthier patients being treated surgically and older, with less active patients being managed nonoperatively. This age difference may have also contributed to some of the differences we saw in terms of union and functional status. Additionally, radiographic union was determined by viewing radiographs taken at routine follow-up appointments; this may not necessarily represent the exact length of time until radiographic union. This study was also limited by incomplete data due to inconsistencies in documentation and variability in follow-up intervals. Due to the retrospective design of the study and the nature of the chart review, we were unable to determine the indication for operative versus nonoperative intervention for every patient. However, the study was adequately powered to assess the two primary outcome measures of time to radiographic union and time to WBAT. Additionally, this study was performed in a single healthcare system, and the patient population may not be representative of or generalizable to the general population. The demographics of the study population are consistent with those that have been previously reported, though, and include urban and suburban populations as well as Level I through non-trauma hospitals [21-23].

Larger prospective studies are needed to address many of these limitations, aid in further examining time to union, and better assess functional outcomes with tools such as patient-reported outcomes measures. Despite the limitations outlined above, this study demonstrates that patients with humeral shaft fractures treated operatively experience a shorter time to radiographic union and weight-bearing than patients treated nonoperatively, without increased complications. This may allow these patients to return to work and activity earlier and contribute to an overall lower cost to society; however, further studies are needed to examine this fully.

## Conclusions

Operative treatment of humeral shaft fractures results in significantly less time for radiographic union and weight-bearing; this may indicate a faster return to work and activities as well as improved functional outcomes.
